# The Dynamical Asymmetry in SARS-CoV2 Protease Reveals the Exchange Between Catalytic Activity and Stability in Homodimers

**DOI:** 10.3390/molecules30071412

**Published:** 2025-03-22

**Authors:** Velia Minicozzi, Alessandro Giuliani, Giampiero Mei, Leonardo Domenichelli, Mauro Parise, Almerinda Di Venere, Luisa Di Paola

**Affiliations:** 1 INFN and Department of Physics, University of Rome Tor Vergata, 00133 Rome, Italy; minicozzi@roma2.infn.it; 2 Department of Environment and Health, Istituto Superiore di Sanità, 00161 Rome, Italy; alessandro.giuliani@iss.it; 3 Department of Experimental Medicine, University of Rome Tor Vergata, 00133 Rome, Italy; mei@med.uniroma2.it; 4 Unit of Chemical-Physics Fundamentals in Chemical Engineering, Department of Science and Technology for Sustainable Development and One Health, Università Campus Bio-Medico of Rome, 00128 Rome, Italy; l.domenichelli@alcampus.it; 5 Unit of Electrotechnics, Department of Engineering, Università Campus Bio-Medico of Rome, 00128 Rome, Italy

**Keywords:** molecular dynamics (MD) simulation, main protease (M^Pro^), SARS-CoV-2, structural analysis, drug discovery, therapeutic interventions

## Abstract

The molecular approach to understanding the mechanisms of emerging diseases, like COVID-19, has largely accelerated the search for successful therapeutical strategies. In this work, we present an extensive molecular dynamics (MD) analysis of two forms of the SARS-CoV-2 main protease M^Pro^. We analyzed the free form (apo) and compared the results with those coming from the (holo) form bound to the inhibitor Boceprevir, an FDA-approved drug repurposed for COVID-19 therapy. We applied Dynamic Cross Correlation (DCC) analysis to the MD simulations to trace the concerted motion patterns within the protein structure. Although symmetric, the homodimer in the bound form showed clearly asymmetric dynamical behavior. In particular, the presence of concerted motions was detected in the protomer where the expulsion of the substrate from the active site happened. Such behavior was not observed in the same time lapses in the apo form. These results highlight a sort of ‘symmetry breaking’, making a symmetric structure to display functional induced asymmetric behavior in response to a perturbation. This highly coordinated dynamics in response to an external cue confirms the character of ‘complex molecular machines’ of biopolymers.

## 1. Introduction

As the outbreak of the novel coronavirus disease (COVID-19) caused by severe acute respiratory syndrome coronavirus 2 (SARS-CoV-2) hit with an unprecedented global impact, it was evident a better understanding of molecular mechanisms behind virus infection was a winning strategy to reach an efficient therapy [1,2].

The main viral protease (M^Pro^), also known as 3Cl^Pro^, is one of the key targets for drug development against SARS-CoV-2 [3]. This enzyme plays a crucial role in viral replication and is therefore an attractive target for therapeutic interventions.

The M^Pro^ protease appears to be an attractive pharmacological target due to its essential role in processing polyproteins translated from viral RNA [4]. This protease operates at no fewer than 11 cleavage sites on the large polyprotein 1ab. It is a dimer composed of two identical subunits that together form two active sites. Inhibiting the activity of this enzyme would block viral replication. Moreover, since no known human proteases exhibit a similar cleavage specificity, such inhibitors are unlikely to be toxic to humans. For this reason, numerous studies have therefore focused on the search for potential inhibitors [4,5,6]. Scientific research has also focused on identifying molecules that could prevent its dimerization. It was, in fact, discovered that in solution, this protease does not perform its enzymatic function when present as a monomer [7,8,9]. A very recent study demonstrated that the M^Pro^ dimer in complex with peptides mimicking the substrate adopts an asymmetric structure: the binding of substrate to a single subunit reveals allosteric communication between the active sites. Furthermore, arginine 4 and 298 are found to be crucial factors in the transition from a symmetric to an asymmetric dimer [10].

**Figure 1:**
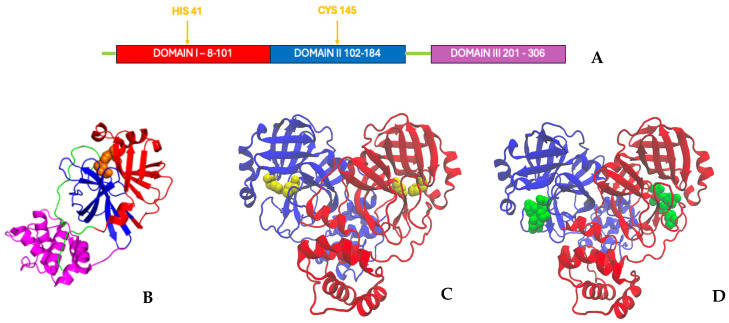
Pictorial representation of the domains of M^Pro^: (**A**) domain distribution along the sequence of the monomer; (**B**) domains in the ribbon structure of the single chain; (**C**) domain spatial distribution in the apo dimer (in blue and red the two subunits); in yellow, the catalytic site residues (HIS 41 and CYS 145, pdb file 6y2e); (**D**) domain spatial distribution in the holo dimer with the inhibitors reported in green (pdb file 7brp).

From a structural point of view, each monomer subunit, which comprises three different domains (I, II and III), is unstable [7]. Dimerization is promoted by domain III aggregation (see Figure 1) and the oligomeric form is stabilized by the tight binding between the C and N terminus of the two chains and by the salt bridge between Glu290 of one chain and Arg4 of the second chain [11]. Since the dimerization is a key step in the catalytic function of the M^Pro^, targeting the dimer interface is a potentially winning strategy to reduce the viral infection [12].

The protein functional region resides (in each chain) at the intersection between domains I and II and it is characterized by a catalytic dyad formed by His41 and Cys145, which is strongly conserved among coronaviruses. M^Pro^ binding is allosterically regulated and different allosteric sites have been identified on its surface. Alzyoud and coworkers [13] detected two classes of M^Pro^ allosteric sites: distal and dimerization sites. Binding to these sites of potential allosteric inhibitors has been demonstrated to alter the catalytic function through conformational changes that involve both subunits. In SARS-CoV M^Pro,^ the co-operation of the two monomers resulted in being quite peculiar and, in fact, functional asymmetry was detected by Chen and co-workers in 2003 [14]. Such a feature was later confirmed for SARS-CoV2 M^Pro^ by Iida et al. in 2021 [15].

Molecular dynamics (MD) is the key computational technique to describe the protein function through its dynamics. Starting from the knowledge of atoms dynamics according to classical force fields, it allows gaining insights about the structure–function relationship in protein molecules, the protein–protein and protein–ligand interactions [16,17,18,19,20]. Since the outbreak of the COVID-19 pandemic, molecular dynamics (MD) has been applied to the molecular components of SARS-CoV-2, specifically the spike protein and M^Pro^, to explore molecular mechanisms and develop viable and effective therapeutic strategies [2,14,21,22,23,24,25,26,27,28,29,30].

In this work, we move forward by providing a two-fold methodology for the punctual analysis of M^Pro^ MD simulations so as to identify crucial regions and residues for the enzyme activity. We applied the analysis to the MD simulations of M^Pro^ (free and bound to the inhibitor Boceprevir, an FDA-approved drug for COVID-19 therapeutical applications [31]). Boceprevir is a protease inhibitor originally used to treat hepatitis C [32] and it has been investigated against SARS-CoV-2 because of the following: (1) SARS-CoV-2 protease shares structural similarities with the HCV protease, which is the target of Boceprevir; (2) in vitro studies have shown that Boceprevir can bind to the active site of M^Pro^, preventing the maturation of viral proteins [33]; and (3) since Boceprevir is already approved for human use, it has a well-known safety profile, shortening the timeline for drug repurposing.

In this way, we were able to compare the dynamics of apo and holo (inhibited) forms and so identify a dynamical signature of inhibition. This approach allows defining the properties of good candidates for COVID-19 therapy targeting M^Pro^.

The method we applied is the computation of the Dynamical Cross Correlation (DCC) matrix based on single-residue trajectories along with MD simulations, while at the same time giving some theoretical hints on the character of biopolymers as complex systems. The Pearson correlation coefficient between the trajectories of residue pairs allows detecting concerted residue motions by tracing out the allosteric pathways in a dynamic way [34,35,36].

The identification of communication pathways in M^Pro^ plays a pivotal role in the comprehension of its action and in the identification of allosteric inhibitors [13,30]. Understanding collective motions aids in comprehending how different regions of a protein communicate during its function and how to disrupt this communication. Thus, findings from this study may have significant implications for developing effective therapeutic strategies targeting this vital enzyme in SARS-CoV-2.

## 2. Materials and Methods

### 2.1. Structure Identification

We applied the analysis on two molecular structures of M^Pro^ from the RCSB PDB server.

The PDB file of the apo protein was generated from the mmCIF file available in the Protein Data Bank (6y2e). Specifically, we used the mmCIF file corresponding to the biological assembly (i.e., a homodimer protein) from the PDB server https://www.rcsb.org (access 10 January 2024). The server https://mmcif.pdbj.org (access 10 January 2024) was used to convert the mmCIF format to PDB format. This server uses, for the conversion, the MAXIT tool from the RCSB PDB server.

For the complex with Boceprevir, we used the 7BRP pdb file.

### 2.2. Molecular Dynamics Simulations

Classical MD simulations were performed by employing the open-source software GROMACS 2021.7 [37] with the CHARMM36 force field [38]. We built two different systems, one for the apo M^Pro^ and one for M^Pro^ in complex with Boceprevir. The ligand (Boceprevir) force field was computed with CGenFF version 2.5 [39,40,41] and translated to be used in GROMACS with the help of a python script (cgenff_charmm2gmx.py) by Justin Lemkul downloaded from Github. Each of the systems was placed in a cubic box with a side equal to 10 nm and we considered the presence of periodic boundary conditions. Each box was filled with TIP3P water molecules and an appropriate number of counterions necessary for the system neutrality (0.15 M). The simulation procedure was as follows: after the minimization, which was performed with the steepest descent algorithm, we equilibrated each system for 1 ns first in the NVT and then in the NPT ensemble. The successive production runs each consisted of 300 ns long simulation in the NPT ensemble at 310 K, by employing the v-rescale thermostat [19], with a coupling time of 0.1 ps. The pressure was kept constant at 1 bar, with a coupling time of 2 ps, and an isothermal compressibility of 4.5·10^−5^ bar^−1^ by means of the Berendsen barostat [42]. The bonds involving hydrogen atoms were constrained according to the LINCS algorithm [43]. The radius of the sphere, which defines the list for the pairwise interactions, was set to 1.0 nm. Consistently, the van der Waals interaction was also cut off at 1.0 nm. The particle mesh Ewald method [44] for the long-range electrostatic interactions was employed using a 1.0 nm real-space cutoff. A time step of 2 fs was used to integrate the equations of motion.

Three distinct simulations were performed for the two forms. The MD data analysis was carried out with standard GROMACS tools. The MD simulations were performed on LEONARDO GPU cluster at CINECA.

### 2.3. Molecular Dynamics Analysis

#### 2.3.1. DCC Analysis

We applied a Canonical Analysis of Motion to the MD frames to identify concerted motions of protein regions [45].

At first, we computed the Displacement Matrix (*DISPL*), an *m × p* matrix, *m* being the number of frames and *p* the number of residues: its *i*-th row contains the displacement values (Euclidean distances between the alpha carbons of the same residue at different times) for all residues at time $t_{i}$ with respect to $t_{i-1}$. The first row was calculated with respect to the cystallographic PDB structure.

The *DISPL* columns report the single-residue displacement along time, somehow representative of the single-residue trajectory.

Once the *DISPL* is computed, it is possible to compute the Dynamic Cross Correlation (DCC) matrix, whose generic element $DCC_{ij}$ is the Pearson correlation coefficient between the displacement vectors of the *i*-th and *j*-th residues, computed as follows:

(1) $$\mathrm{DCC}\left(i,j\right)=\frac{\langle \Delta r_{i}\left(t\right)\cdot \Delta r_{j}\left(t\right)\rangle }{\sqrt{\langle \|\Delta r_{i}\left(t\right){\|}^{2}\rangle \;}\cdot \sqrt{\langle \|\Delta r_{j}\left(t\right){\|}^{2}\rangle }}$$

where $r_{i}\left(t\right)$ represents the i-th residue alpha-carbon position as a function of time while $\Delta r_{i}\left(t\right)\;$ is the residue displacement in the frame at *t* (generic element of the matrix *DISPL*). DCC allows for a deep understanding of the MD simulations, revealing the concerted motions between distal residues [2,46,47]. In other words, if the correlation between the displacement of the two vectors for the two residues is high, that means those two residues move in a concerted motion. So, a high value of ${\mathrm{DCC}}_{ij}$ means that residues *i*-th and *j*-th move together.

**Figure 2:**
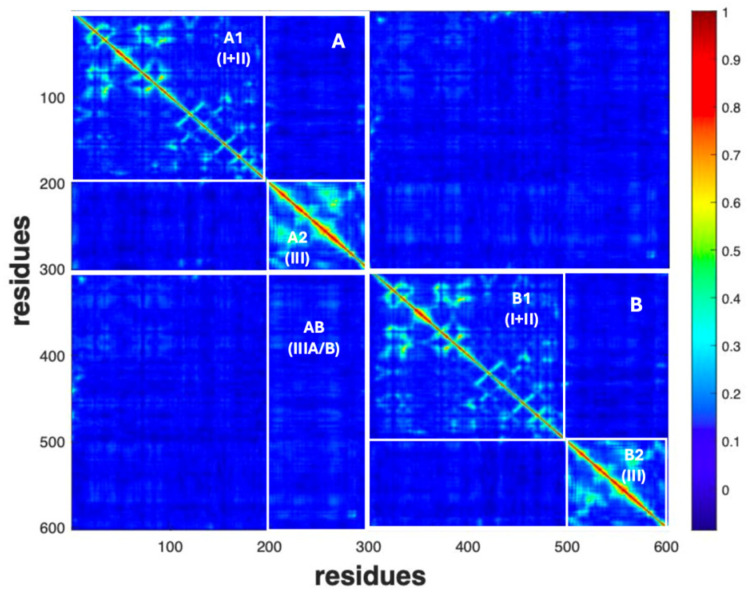
Identification of regions in the DCC maps (for the apo form) to better highlight the concerted motions between and within domains and chains. A1 and B1 refer to the region including domain I + II in both chains, whereas A2 and B2 comprise domain III in the chains. A and B comprise single chains. Region AB includes interactions between domain III of chain A and chain B.

To better emphasize the coordinated movements within and between different regions of the protein M^Pro^, we identified specific areas in the dynamic correlation map (DCC map) based on unique findings, as depicted in Figure 2. The color scale reports the value of the DCC in terms of hot (cold) colors corresponding to high (low) values of correlation coefficients between a pair of residues (hot values → high probability of concerted motions, cold values → low probability of concerted motions). So, the large blue regions correspond to a pair of residues whose displacement vectors are completely uncorrelated (correlation coefficient ~0. On the other hand, red spots correspond to residues with highly correlated (concerted) motions, for instance, residues in the same alpha-helix.

For this analysis, we grouped domain I + II together (A1 and B1 in the two chains, respectively), as they are responsible for the catalytic activity of M^Pro^, and considered them separately from domain III (A2 and B2). Region AB comprises the interactions between domain III of chain A and chain B, emerging as dynamically correlated.

Finally, we also considered motions within the single chains (A and B in Figure 2).

#### 2.3.2. Protein Contact Network Analysis

The protein contact networks are obtained from molecular dynamics frames (pdb files), according to the method widely explained in a previous paper [48]. In brief, for each frame, the alpha-carbons’ positions are extracted and the adjacency matrix of mutual distances between residues is computed. A link exists between two residues if their distance falls between 4 and 8 Å. The adjacency matrix is the descriptor of the protein contact network; it is a binary matrix whose generic element is one if there is a link between the corresponding nodes. From matrix A, it is possible to extract several descriptors and in particular the betweenness centrality (*btw*). The *btw* is a protein contact network descriptor that measures the importance of a residue in mediating communication between other residues in the network. Specifically, it measures the number of shortest paths that pass through a given residue according to the following equation:

$$btw\left(i\right)=\underset{v\in V,\;v\neq i}{\sum }\underset{u\in V,u\neq i}{\sum }\frac{\sigma_{uv}\left(i\right)}{\sigma_{uv}}$$

where $\sigma_{uv}$ is the number of shortest paths connecting nodes *u* and *v* and $\sigma_{uv}\left(i\right)$ is the number of shortest paths connecting the two nodes through node *i*.

## 3. Results

**Figure 3:**
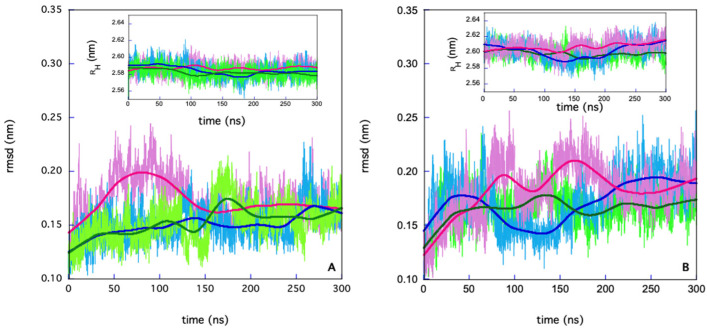
RMSD for the three simulations for the apo (**A**) and the holo (**B**) forms. In the insets, the trends of the protein’s radius of gyration as a function of time are shown for all simulations. The bold lines represent the result of curve fits obtained using a smoothing factor of 20% through the center of the data (Kaleidagraph Sinergy software 5.0).

Figure 3 reports the RMSD for the three simulations for the apo and holo forms. In the insets, the corresponding evolution of the protein’s radius of gyration (R_H_) is reported.

The three simulations for the holo form start from a condition of double occupancy of the catalytic sites. All systems reached stability in the last 100 ns of the MD simulations as demonstrated by the standard deviations value of the RMSD and R_H_ calculated in the range (200–300) ns (less than 8%).

On the basis of the events that take place at the inhibitor binding sites, the MD time course can be divided into different phases.

**Figure 4:**
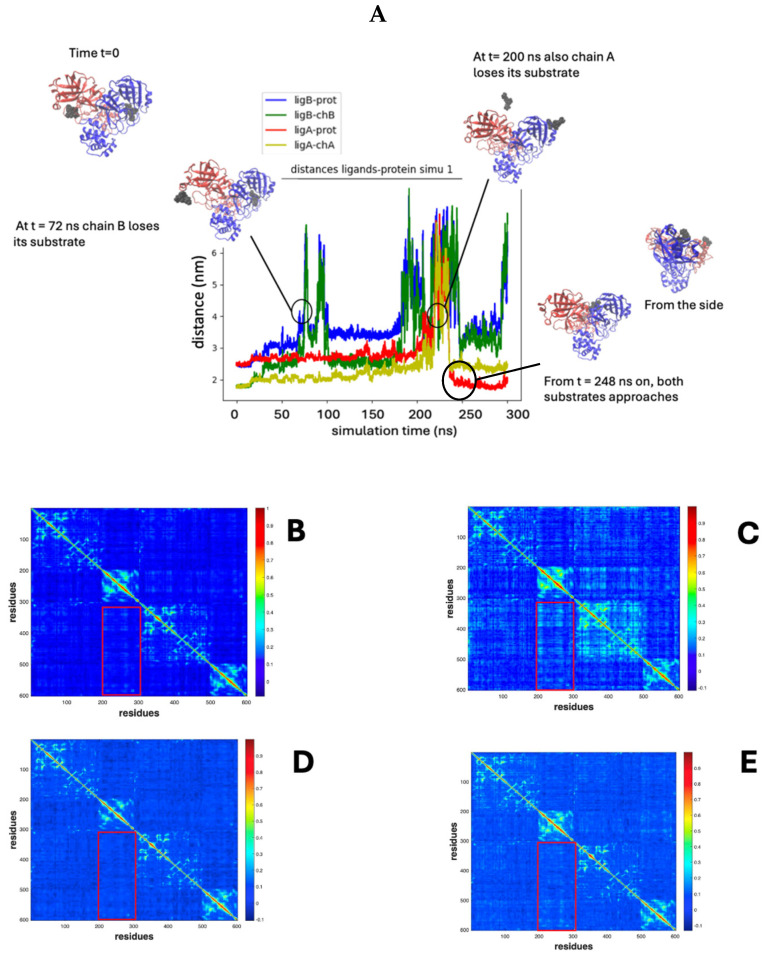
(**A**) Simulation 1 of the MD of the holo form: the figure reports the distance between substrates and their corresponding catalytic phases. Three phases are detected, corresponding to the asymmetric occupancy of the two catalytic sites. (**B**) DCC for the whole MD simulation. (**C**) DCC for phase I. (**D**) DCC for phase II. (**E**) DCC for phase III. In red rectangles, the AB region of the map, reporting data of correlation between motions of residues in domain III of chain A and chain B.

For instance, in Figure 4, the case of holo simulation #1 (Figure 3B, green) has been quantitatively analyzed by evaluating the distances between the two inhibitor molecules and the two protein subunits. In particular, an asymmetric dynamic behavior can be observed and three different MD phases identified (Figure 4A) as follows:

1. PHASE I: in the first 72 ns, both chains retain their substrates; at t ≈ 72 ns, chain B loses its substrate;
2. PHASE II: from t ≈ 72 ns to 200 ns, only chain A retains its substrate; at t = 200 ns, chain A also loses its substrate;
3. PHASE III: from t = 200 ns, both chains are unbound.

In order to characterize the details of the inter-subunit interaction along the simulation time course, the DCC matrix has been evaluated and the profiles for the whole simulation time and the three individual stages are shown in Figure 4B–E. The first phase (stage I, Figure 4C) is marked by a general increase in correlated movements between individual residues, suggesting a high degree of coordination across the protein structure. Following substrate 2 detachment in stage II, there is a reduction in the correlation, indicative of reduced concerted motion (Figure 4D).

In stage III (Figure 4E), there is a general reduction in the correlation, approaching the values of phase I. This pattern suggests a dynamic process where initial coordinated enzyme “shaking” facilitates the first substrate ejection (in chain B), followed by a quieter phase, and then a partial resurgence of correlated movements, associated with the release of the second substrate molecule (from chain A). Notably, the AB region displays weak but significant correlations in stage I, which disappear in stage II and reappear, though fainter, in phase III, highlighting its potential role in substrate interactions. 

**Figure 5:**
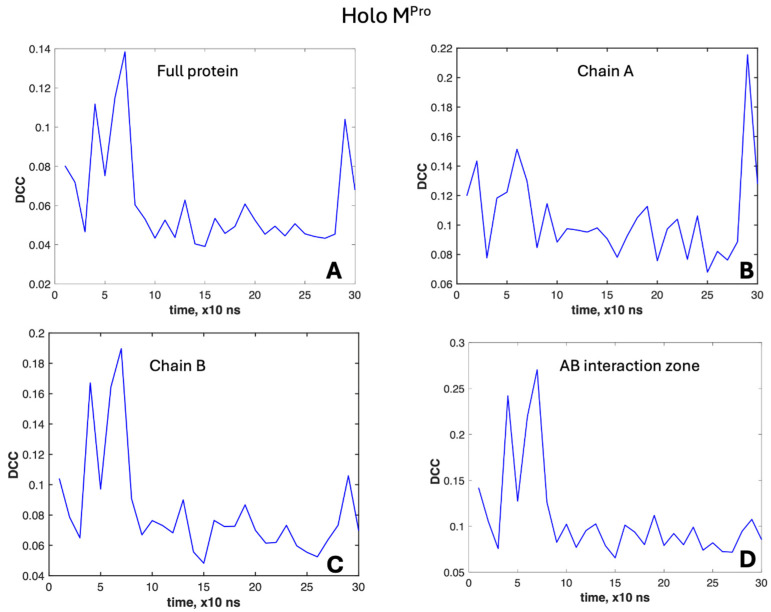
DCC variation in molecular dynamics of the holo form of M^Pro^. (**A**) Whole molecule. (**B**) Chain A. (**C**) Chain B. (**D**) Interaction zone AB.

**Figure 6:**
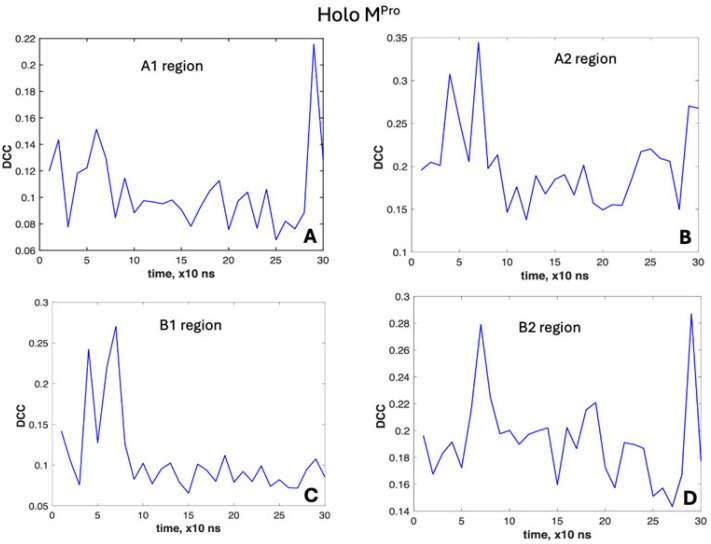
DCC variation in molecular dynamics of the holo form of M^Pro^. (**A**) Region A1. (**B**) Region A2. (**C**) Region B1. (**D**) Region B2.

To better characterize these dynamics, we applied the DCC method over a time range of 10 ns (100 frames). The results are reported in Figure 5 and Figure 6.

Figure 5 reports the DCC’s time course for the whole holo molecule, the two chains and the region of interaction (AB in Figure 2).

The whole protein, chain B and region AB experience two peaks of correlation around 40 and 70 ns, the latter being the highest value (0.18 for chain B and 0.27 for the AB region).

Going more in detail (Figure 6), the regions experiencing these two peaks are A2 (reaching at 70 ns the highest value of 0.34, Figure 6B) and B1 (Figure 6C). Meanwhile, B2 exhibits a lower peak at 40 ns and a noticeable peak at 70 ns (0.28, Figure 6D). Region A1 shows similar peaks, but at quite lower values of the correlation coefficient at different times (0.14 at 20 ns and 0.15 at 60 ns, Figure 6A).

We verified similar patterns for two additional MD simulations of the holo form of M^Pro^ (see Supplementary Files).

Despite the great qualitative impact, the DCC representations in Figure 5 and Figure 6 do not allow a quantitative interpretation of the molecular events in which the protein domains are involved. For such reason, we performed a coarse-graining analysis of such data, calculating the average DCC value (<DCC>) in the different regions identified in Figure 2. In this way, a single DCC value, representing each particular polypeptide region, quantitatively summarizes the temporal correlation of the residues belonging to that area along the simulation.

**Figure 7:**
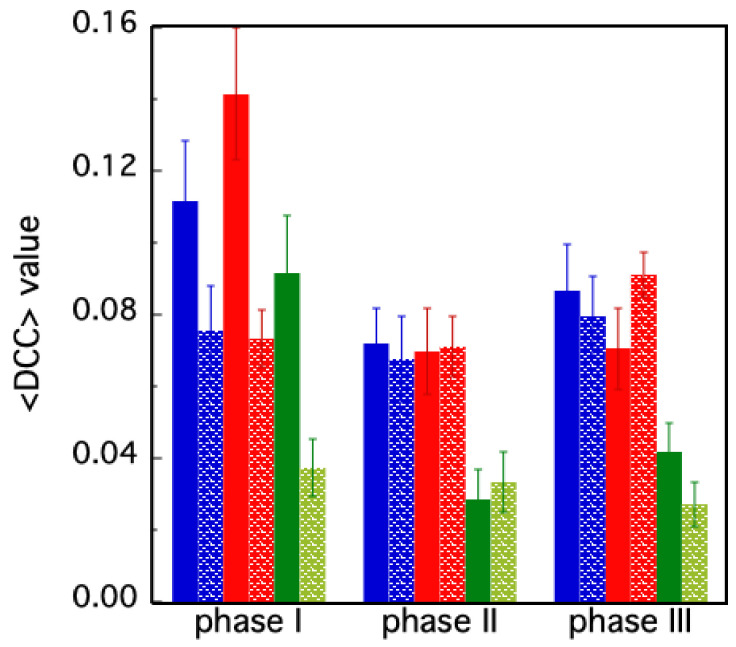
Average DCC values in the different simulation phases for the holo (filled bars) and apo (dotted bars) forms. The blue and red color are associated with chain A and chain B, respectively. The green bars correspond to the <DCC> values evaluated in region AB (see Figure 2). The bars correspond to the standard deviations of the values.

The first result is the quite different behavior of the two chains (A, B), in the holo and in the apo forms. As shown in Figure 7, the <DCC> values in the apo protein are roughly the same in the three phases, oscillating around 0.08. On the contrary, in the holo form, both chains are characterized by higher <DCC> values in the first phase (>0.11). Then, after the release of the inhibitor molecule from chain B, a rapid decrease in the average correlation values is observed in phase II, followed by a slight increase in phase III, involving the sole chain A (in blue, in Figure 7). A second important feature emerging from the <DCC> analysis concerns the AB area of the diagram reported in Figure 2. This region represents the correlation between domains belonging to different protein chains. The high <DCC> value observed in the holo form during phase I (in green, Figure 7) suggests that synchronic motions occur between residues within the catalytic domains of chain B (located in the middle of domains I and II), with those of the oligomerization domain of chain A (i.e., domain III).

**Figure 8:**
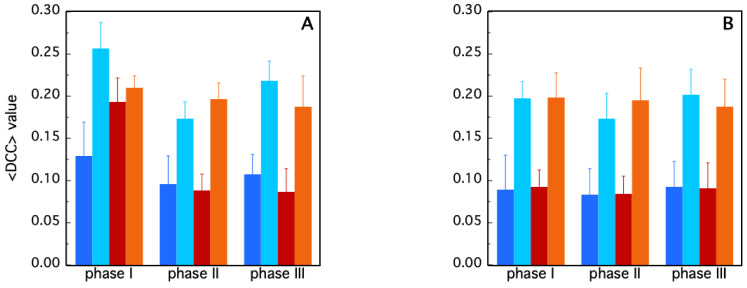
Average <DCC> values evaluated for regions A1 (blue), A2 (cyan), B1 (red), B2 (orange), in the case of the holo (**A**) and apo (**B**) protein.

More details on the intra-domain residues correlation have been obtained by evaluating the <DCC> values for the individual A1, A2, B1 and B2 regions (Figure 2) of both the holo (Figure 8A) and apo (Figure 8B) protein forms.

At first glance, the correlation of the residue trajectories appears very homogeneous only for the apo M^Pro^, which displays marginal oscillation of the <DCC> values as a function of time (Figure 8B). In this case, two striking pieces of evidence emerge as follows: (i) a symmetry between the two chains and (ii) a larger contribution of the A2 and B2 region to the average correlation of each protein chain. This last feature is interesting, since the A2 and B2 areas correspond to domain III (see Figure 2), which is the main domain responsible for the M^Pro^ oligomerization process. High intra-domain correlation values thus suggest that the cooperative organization of the A2 and B2 residues takes place to facilitate the interaction of the two chains at the monomer–monomer interface. The case of the holo protein is more complex. According to the values reported in Figure 8A, regions A2 and B1 play a main role during phase I of the simulation. Then, large changes occur, with the <DCC> values in A2 oscillating from—32% in phase II, to the recovery of about + 20% in phase III. Interestingly, a similar trend occurs in the case of A1, despite it being to a lower extent. Different behavior was instead found for the residues of the B1 region. In this case, we observe a decrease in the <DCC> value after the release of Boceprevir (phase II and III).

In order to elucidate the role of the dimeric interface in constituting the active dimer, we have analyzed the *btw* values of the residues of the holo samples at different MD frames. Residues with high *btw* are important for maintaining the overall connectivity and communication within the protein structure; they often act as “bridges” in transmitting conformational information between different regions of the protein. They are, therefore, prime candidates for involvement in allosteric mechanisms [48].

**Figure 9:**
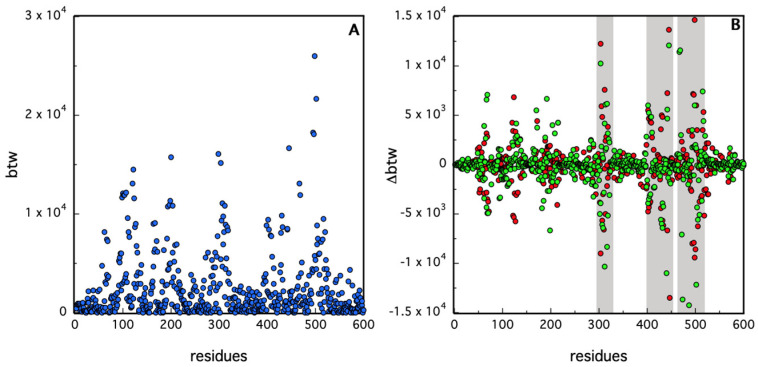
(**A**): *btw* values at 30 ns are reported for residues of holo chain A (1–300) and B (301–600); in (**B**), the difference in bwt values at 150 ns (green) and 240 ns (red) compared to those at t = 30 ns is reported for each residue. The rectangles in gray highlight the regions with the highest values of Δ*btw*.

In Figure 9, we have shown the *btw* values for the amino acids of the holo protease at t = 30 ns, i.e., during phase I of simulation #1 (panel A). In the same figure (panel B), we have reported the changes in this parameter with respect to those obtained at 30 ns at two other MD times (150 ns and 240 ns). In this graph, the most relevant changes occur in some particular residues of chain B, as evidenced by the rectangles in gray. These changes occur when subunit B releases the inhibitor (from phase I to phase II) but the same B residues are also involved when movements of Boceprevir are in subunit A (from phase I to phase III).

## 4. Discussion

Symmetry is an intriguing feature of oligomers. The number of configurations that such proteins may assume is impressive [49,50] and, since the crystal structure of hemoglobin was solved, it was clear that the symmetric arrangement of oligomers has enormous implications in their biological function. Indeed, the first model of allosterism (MWC) was based on symmetry and it is a milestone in the chemistry of protein–ligand interaction [51]. Yet, at the microscopic level, the so-called “induced-fit” concept has introduced the idea that the crystallographic symmetry of oligomeric proteins and enzymes may be broken, once the first ligand/substrate molecule binds one subunit.

The case of homodimers is very peculiar as they represent the simplest form of subunit interaction: each subunit can exclusively interact/control its own mate; thus, there is no chance for those extended conformational changes that characterize the cooperative functional dynamics of oligomers containing more than two subunits. Moreover, the identity between the two protomers generates a global structural symmetry to the dimer. Nevertheless, it has been found that the two equivalent active sites of homo-dimers are rarely both occupied at the same time, a strange behavior that suggests an intrinsic functional asymmetry for these proteins. Pai and coworkers have provided a rationale for such dynamic asymmetry in the case of a homo-dimeric dehalogenase [52,53]. In particular, they demonstrated that the entropic cost of substrate binding in one subunit is counterbalanced by a more disordered state in the other monomer, such “structural information” being transmitted and shared between the two protomers through an allosteric pathway. Such findings indicate that the degree of motional coordination among the residues in the empty subunit differs from that of the substrate-bound monomer. Similar results were also found for the case of SARS 3C-like proteinase where the two protomers of the dimer are asymmetric and only one protomer is active at one time [14].

The analysis of MD dynamics for the two forms of protease in our study highlights a distinct difference between the dynamics in the presence (holo) and in the absence (apo) of a ligand. In the apo form, with both binding sites empty, a symmetrical pattern between the two chains was found (Figure 7). In particular, the two domains comprising the catalytic residues move in a concerted motion region (regions A1 and B1 in Figure 8), while the third domain (A2 and B2), responsible for the protease dimerization, moves on its own. The two chains of the holo form display, instead, a more complex behavior, the evolution of the DCC pattern depending on the presence/absence of a single or both ligand molecules. For instance, during phase I, higher average correlations occur in chain B with respect to chain A (region B1 in Figure 5B and Figure 7). This means that in attempting to clear out the binding site, the residues in chain B cooperate all together until the ligand is expulsed. Interestingly, during such a phase, strong cooperation between the two chains also takes place, as demonstrated by the high <DCC> values observed in the AB region (Figure 7, phase I, green bar). The release of the second ligand molecule, at the end of phase II, is less dramatic and, in fact, the average DCC values of each holo protein domain progressively re-align to those of the apo form (Figure 8). This peculiar behavior of the protease, in which the ligand is released during the simulation but later rebinds to the active site, has also been observed with other inhibitors in the study conducted by Komatsu and collaborators [54]. This finding suggests that the absence of one ligand confers the whole protein with an enhanced conformational dynamic. On the other hand, such behavior is also compatible with the already mentioned concept of “entropic reservoir”: the free subunit can have a regulatory role by means of an entropy compensation mechanism, confirming the crucial functional role played by empty subunits in homo-dimeric enzymes [51,52].

The important role of the dimer interface in constituting the active molecule and transmitting information through the two monomers is also evident from the analysis of the *btw* values (Figure 9). We have seen that in our case, the values of the *btw* of some peculiar residues of chain B change a lot with respect to chain A (Figure 9). Some of these amino acids (ARG 4, SER 10, GLY 11 and GLU 14), as evidenced in Figure 9B, belong to the so-called allosteric site on the distal region of the protein [13]. Identifying and modifying those residues with high *btw* could help alter or engineer allosteric pathways to modulate protein function since several efforts have been performed to identify drugs able to bind both in the catalytic and distal allosteric binding sites [55].

In conclusion, the two identical polypeptide chains of homodimers confer them with the peculiar symmetry observed in the crystallographic models. Such symmetry is retained in the apo form, but it is immediately lost when the dimer interacts with ligands and a “dynamic asymmetry” emerges. This feature is accompanied by a strong inter-chain correlation and appears as a biological strategy to optimize the performance of such enzymatic systems, confirming that initial binding to one active site allosterically perturbs the second one and generates an asymmetric dimer [10].

The two peaks observed at 40 ns and 70 ns in the detailed DCC’s time course can be interpreted as a “tipping point”, i.e., critical thresholds before a dramatic change in the protein structure and function (the loss of the first substrate molecule). This is justified by the fact these events in complex systems dynamics are detected by peaks in variance and correlation [56].

Finally, together with allostery, the intrinsic dynamic asymmetry of homodimers would explain their widespread presence in the protein world [52].

The emergence of a ‘dynamic asymmetry’ from a ‘structural symmetry’ is a signature of the onset of complexity at the molecular level, confirming the peculiar location of biopolymers on the edge between simplicity and complexity physics [57].

## Data Availability

The raw data supporting the conclusions of this article will be made available by the authors on request.

## Supplementary Materials

The following supporting information can be downloaded at: https://www.mdpi.com/article/10.3390/molecules30071412/s1, Figures S1 and S5: Simulation 2 (S1) and 3 (S5) of the MD of the holo form: the figure reports the distance between substrates and their corresponding catalytic phases. Figures S2, S6 and S8: DCC for the whole MD simulation, DCC for phase I, DCC for phase II, DCC for phase III for holo simulations 2 (S2), 3(S6) and apo simulation 1 (S8). Figures S3, S4, S7 and S8: DCC variation in molecular dynamics of the holo form for simulation 2 (S3 and S4) and simulation 3 (S7 and S8).
