# Supplementary material for: The Dynamical Asymmetry in SARS-CoV2 Protease Reveals the Exchange Between Catalytic Activity and Stability in Homodimers
Source: Molecules. 2025 Mar 22;30(7):1412. doi: 10.3390/molecules30071412 (PMC11990344; doi:10.3390/molecules30071412)
Supplement: Supplementary file 1 [file molecules-30-01412-s001.zip › molecules-3465040-supplementary.pdf]

SIMULATION 2

Time t =0

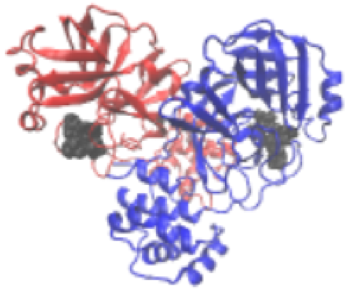

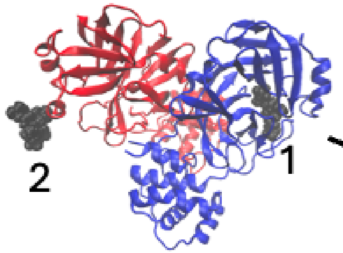

At ≈ 130 ns chain B looses its substrate (2)

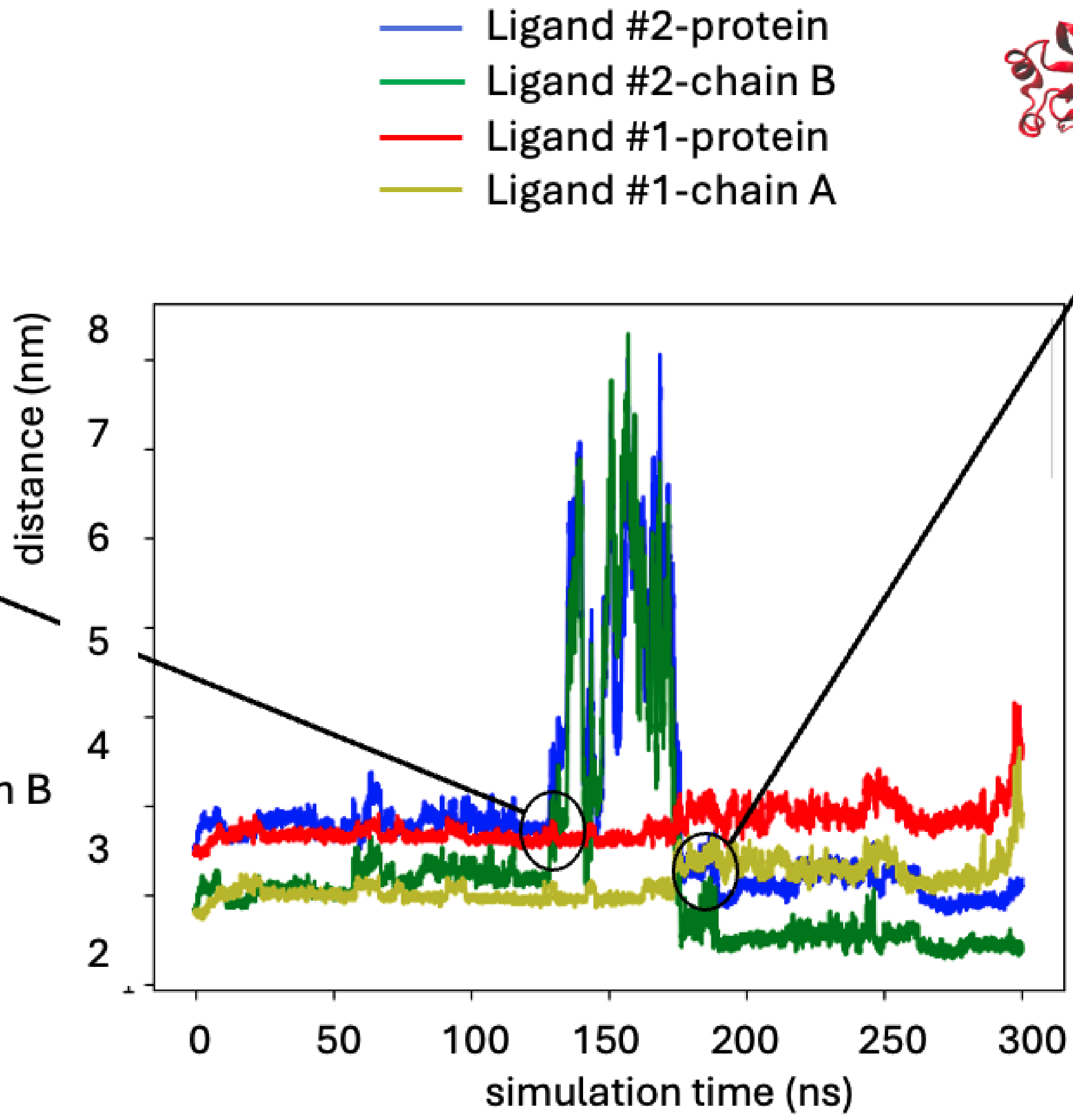

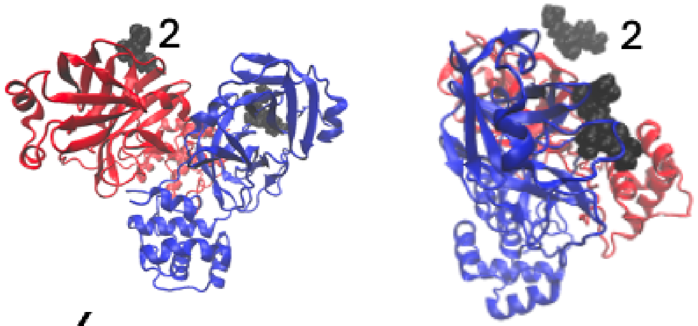

front view side view

At ≈ 175 ns substrate (2) of subunit B approaches back to the protein but not exactly to the catalytic site

In the time course of this second simulation, no detachment of substrate (1) from subunit A was detected

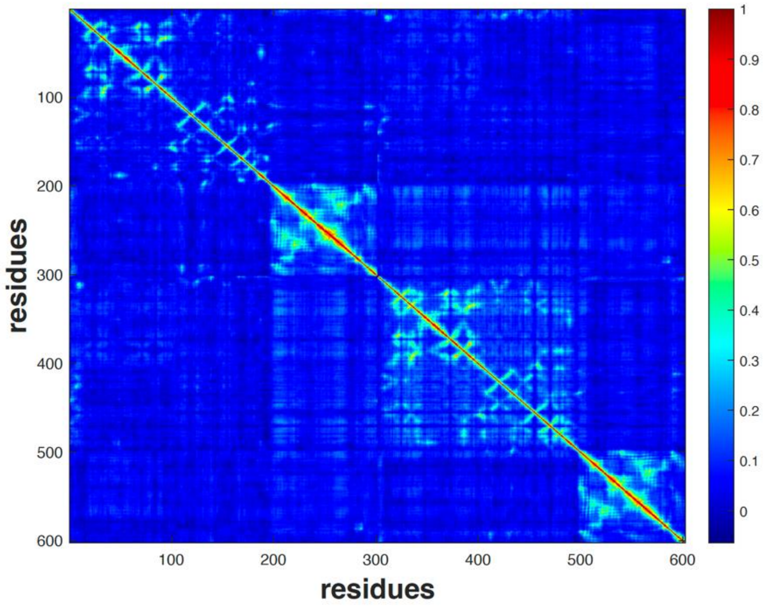

WHOLE

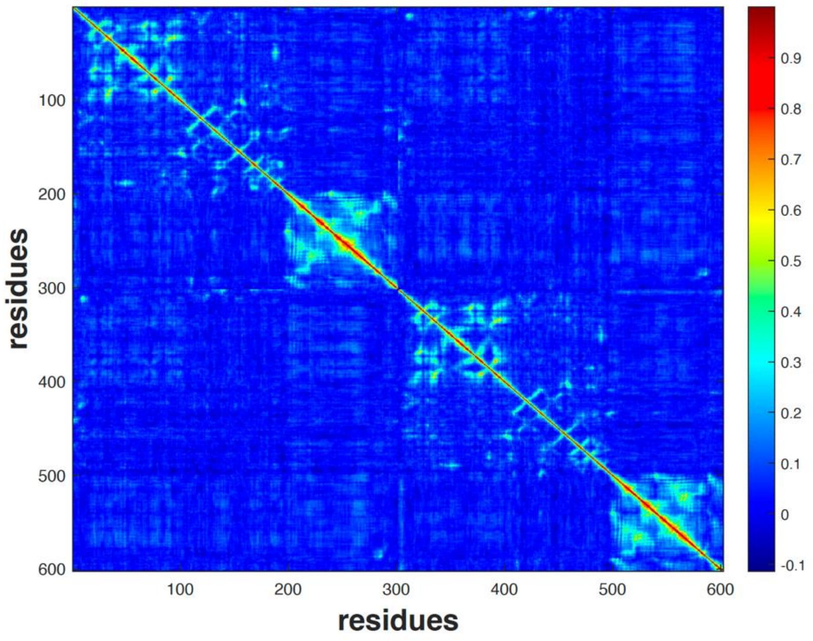

t=0→130 ns sub 1 and 2 ON

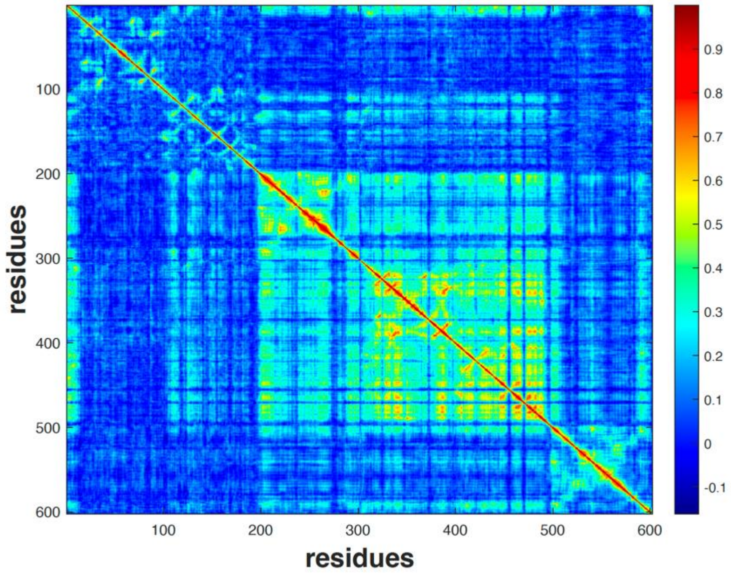

t=130→175 ns sub 1 ON and 2 OFF

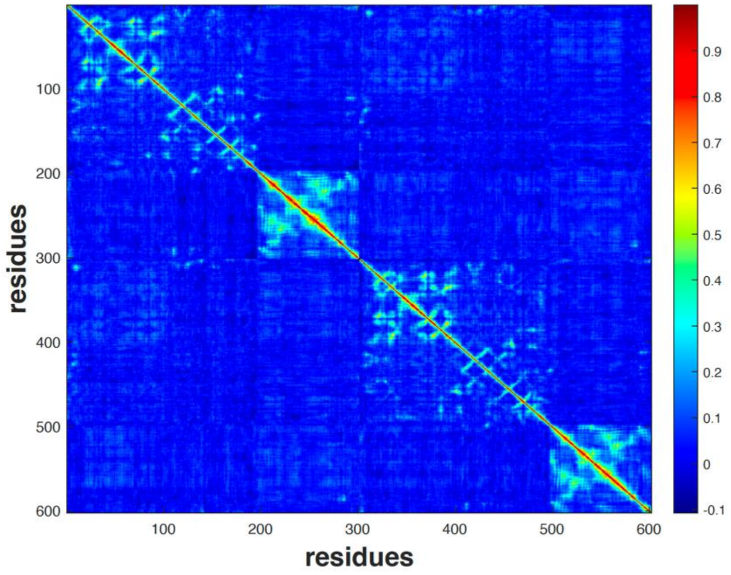

t=175 ns→end sub 1 and 2 OFF (CLOSER)

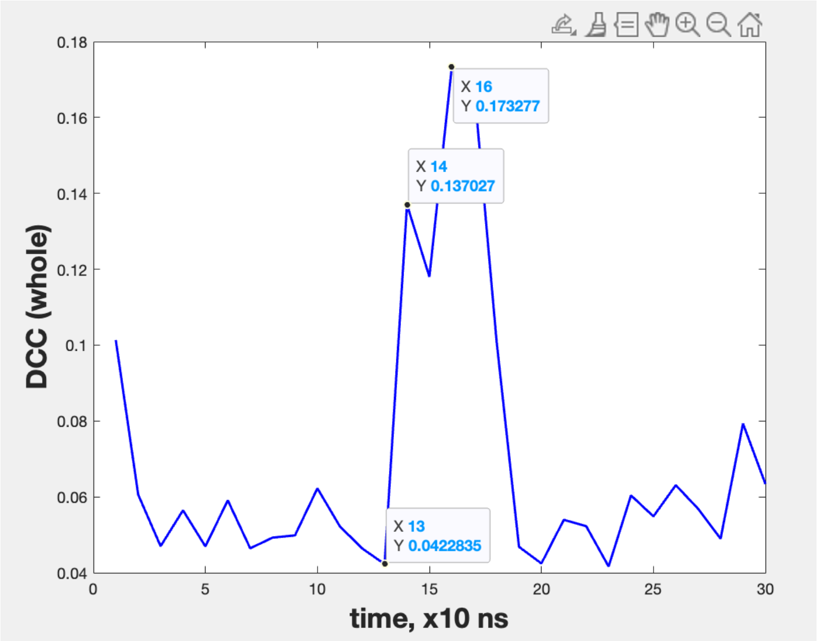
DCC (whole)
time, x10 ns
X 16
Y 0.173277
X 14
Y 0.137027
X 13
Y 0.0422835

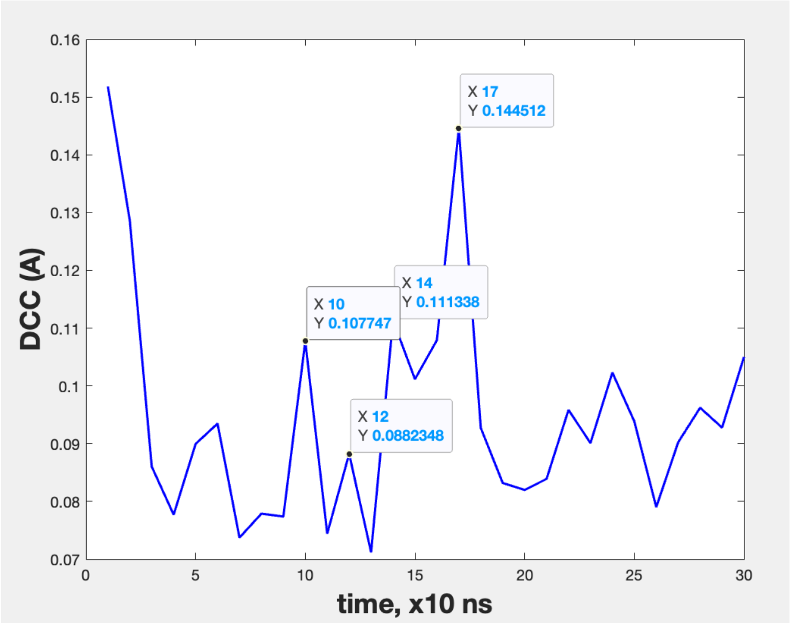
DCC (A)
time, x10 ns
X 17
Y 0.144512
X 14
Y 0.111338
X 10
Y 0.107747
X 12
Y 0.0882348

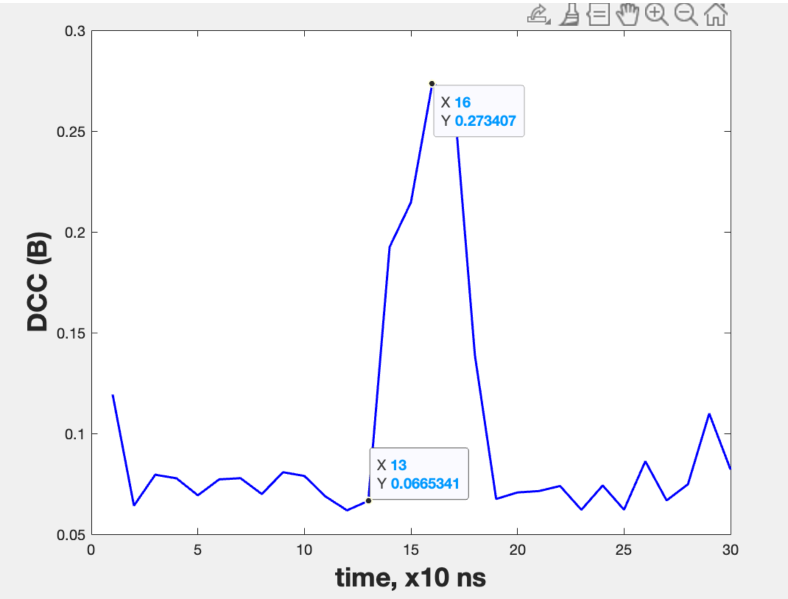
DCC (B)
time, x10 ns
X 16
Y 0.273407
X 13
Y 0.0665341

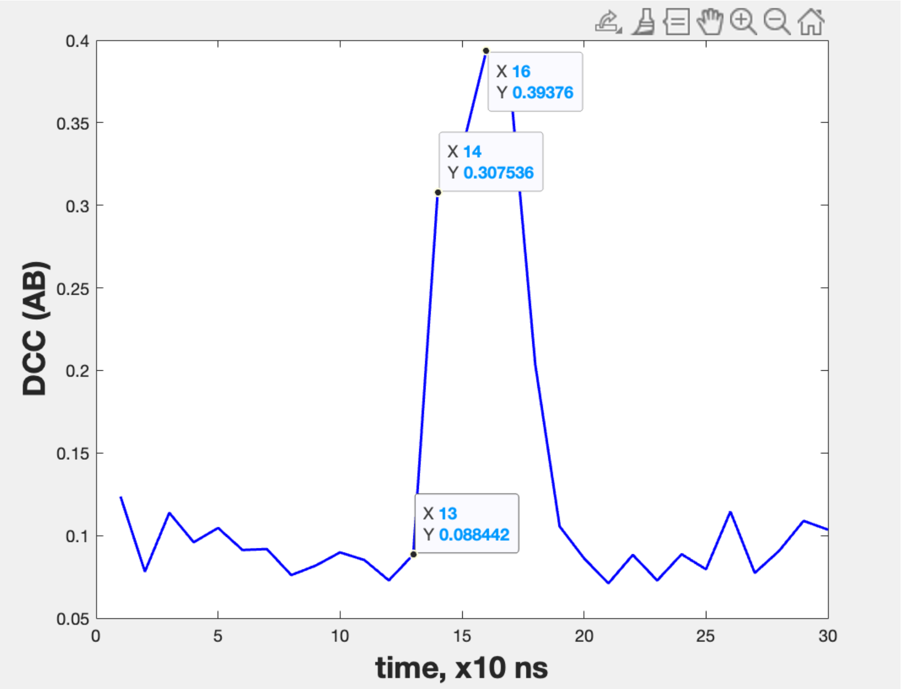
DCC (AB)
time, x10 ns
X 16
Y 0.39376
X 14
Y 0.307536
X 13
Y 0.088442

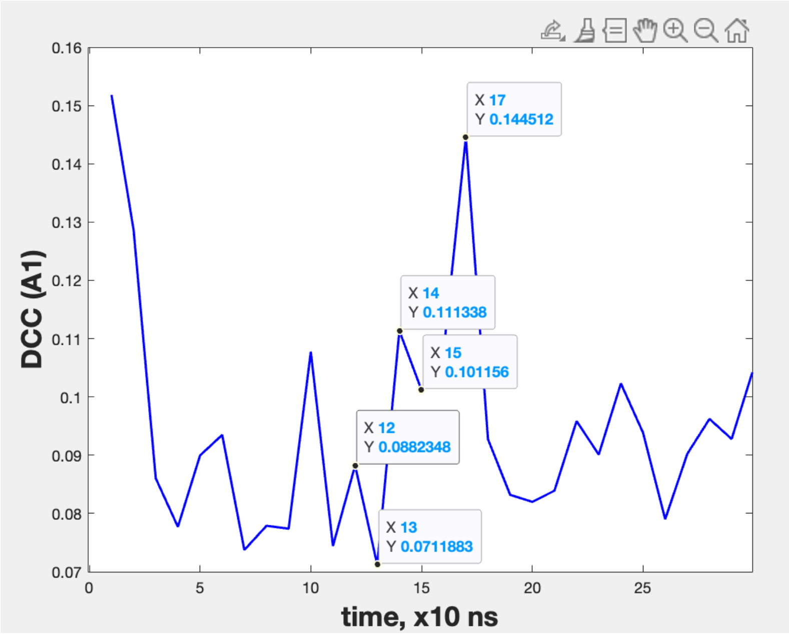
DCC (A1)
time, x10 ns
X 17
Y 0.144512
X 14
Y 0.111338
X 15
Y 0.101156
X 12
Y 0.0882348
X 13
Y 0.0711883

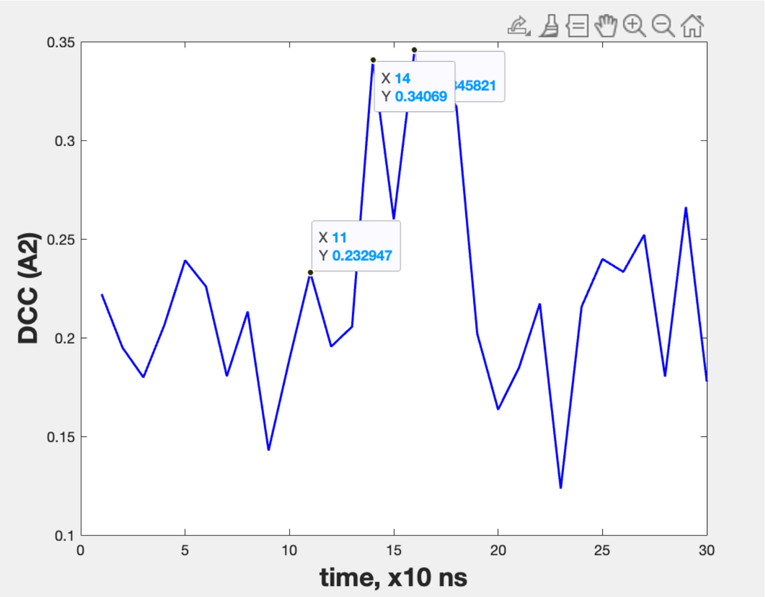
DCC (A2)
time, x10 ns
X 14
Y 0.34069
X 11
Y 0.232947

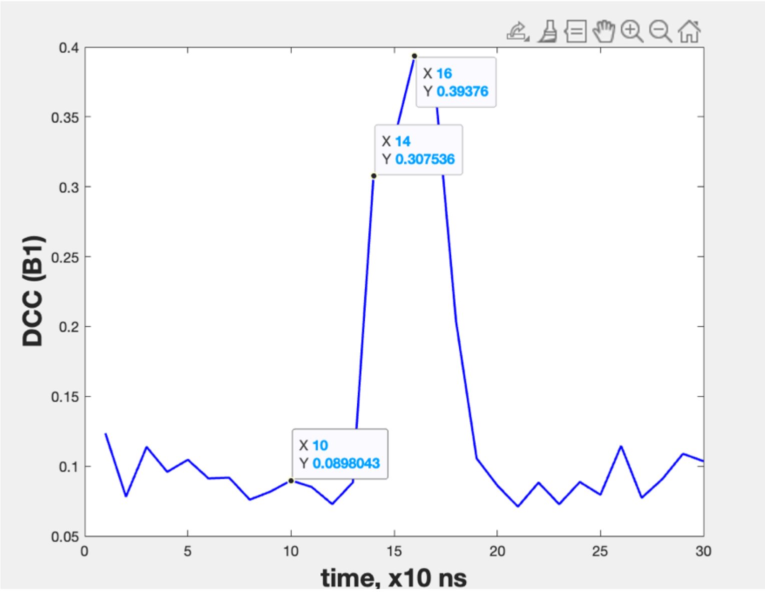
DCC (B1)
time, x10 ns
X 16
Y 0.39376
X 14
Y 0.307536
X 10
Y 0.0898043

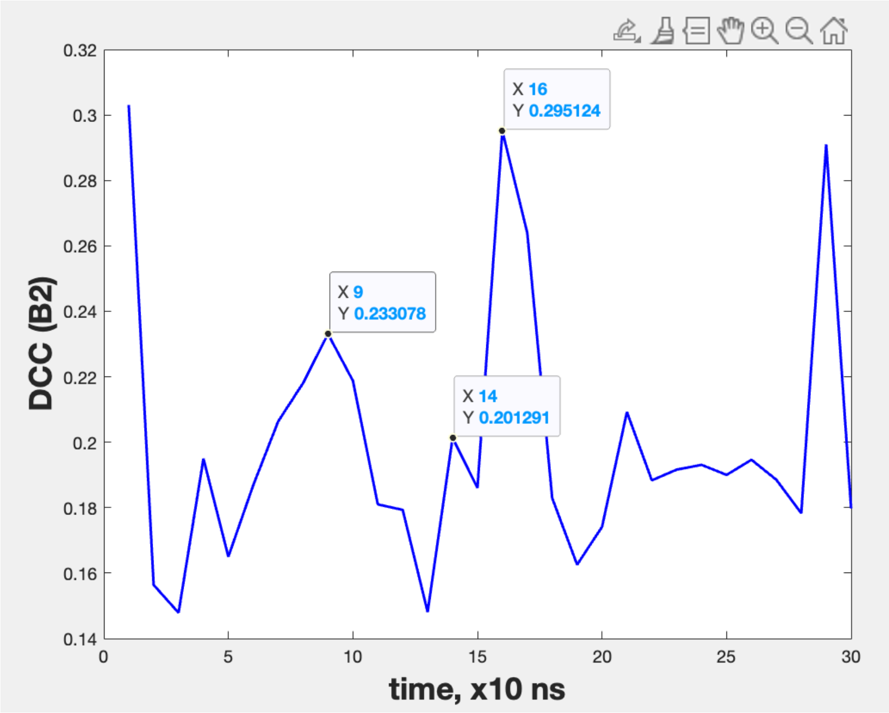
DCC (B2)
time, x10 ns
X 16
Y 0.295124
X 9
Y 0.233078
X 14
Y 0.201291

SIMULATION 3

Time t =0

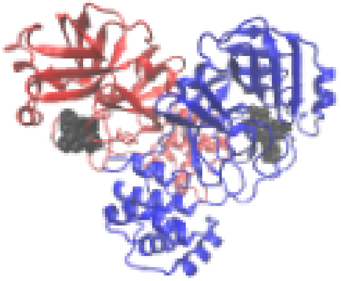

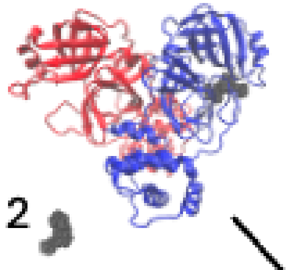

Between ≈ 40 and 70 ns the substrate (2) of subunit B goes back and fort form the active site to solvent

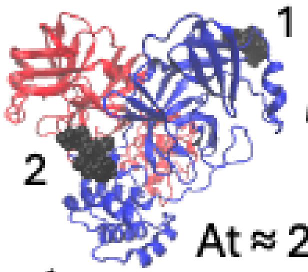

At ≈ 200 ns ligand #1 leaves the active site of subunit A

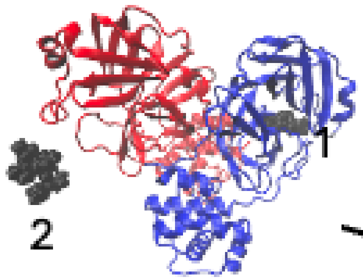

At ≈ 37 ns chain B looses its substrate (2)

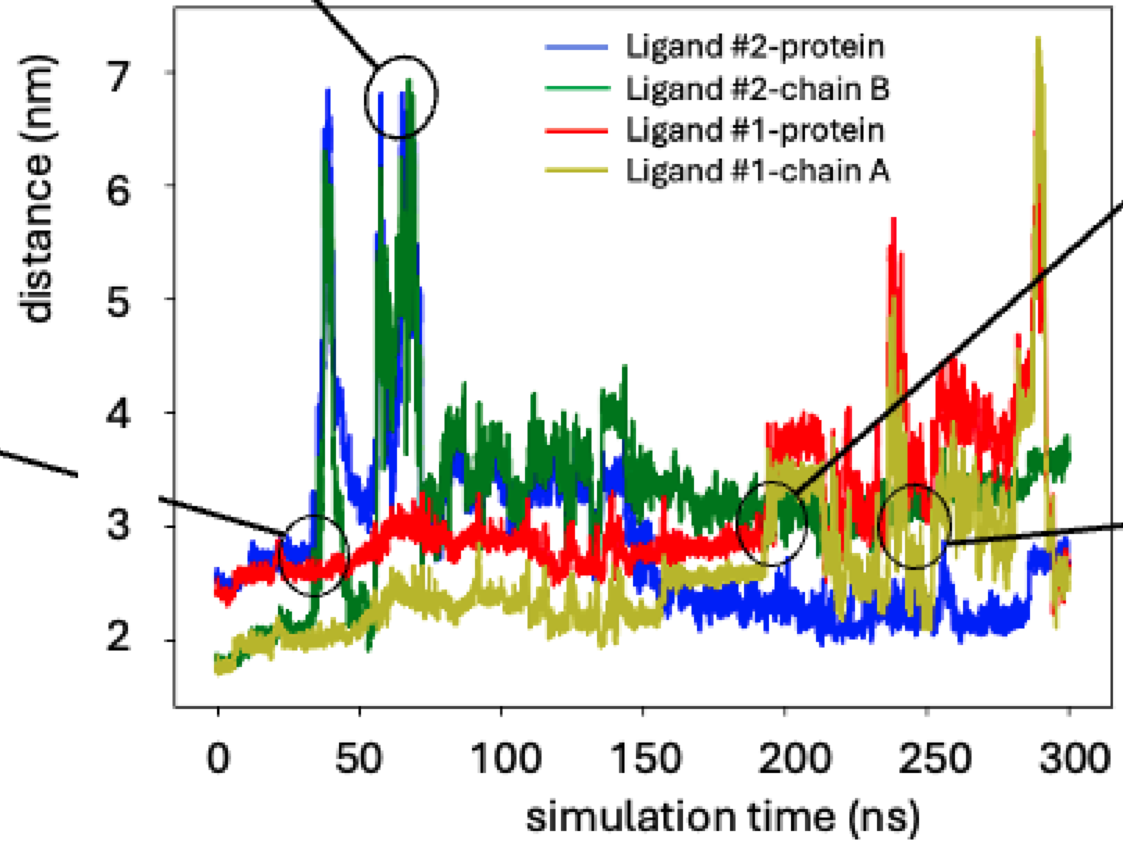

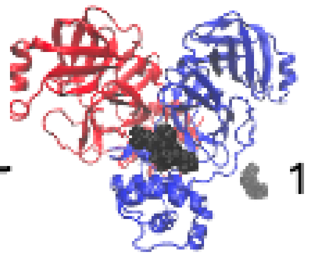

A ≈ 238 ns the distance between ligand 1 and subunit A increases

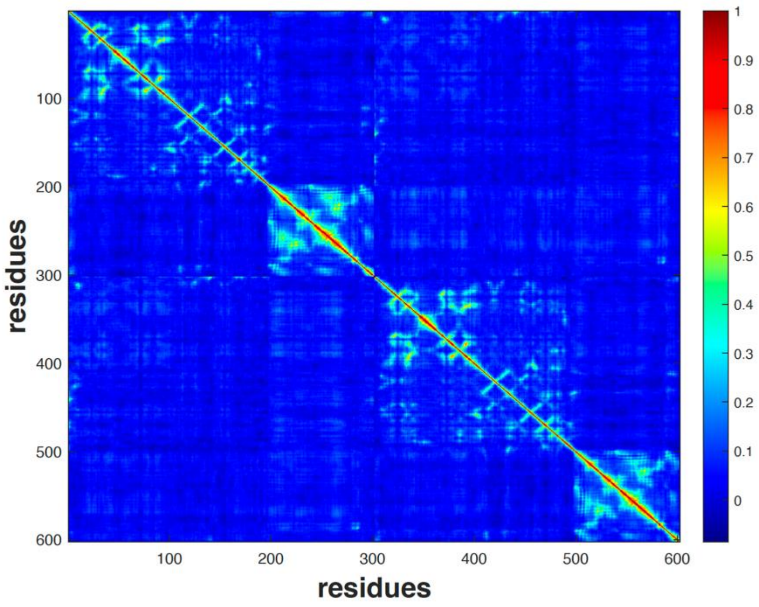

WHOLE

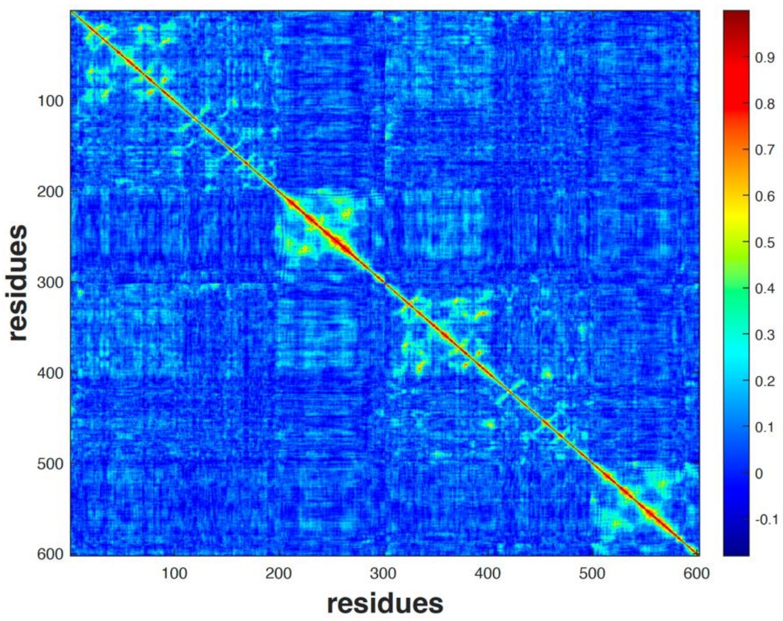

t=0→37 ns sub 1 and 2 ON

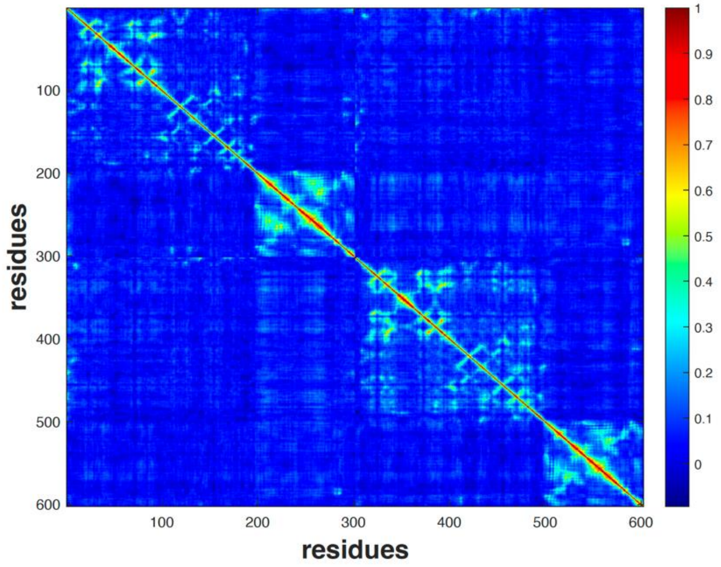

t=37→200 ns sub 1 ON and 2 OFF

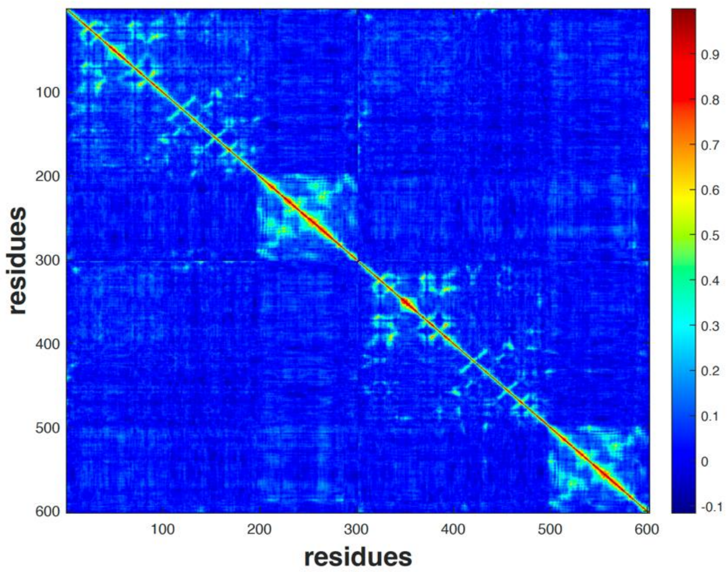

t=200 na→end sub 1 and 2 OFF

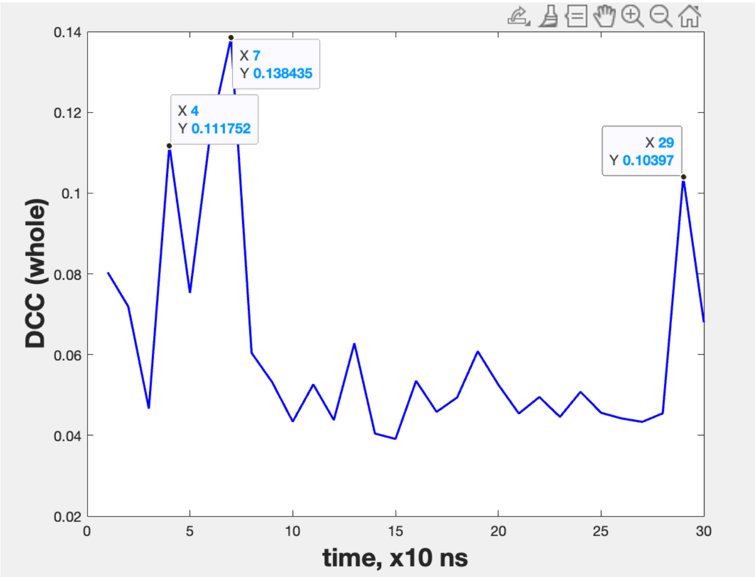
DCC (whole)
time, x10 ns
X 7
Y 0.138435
X 4
Y 0.111752
X 29
Y 0.10397

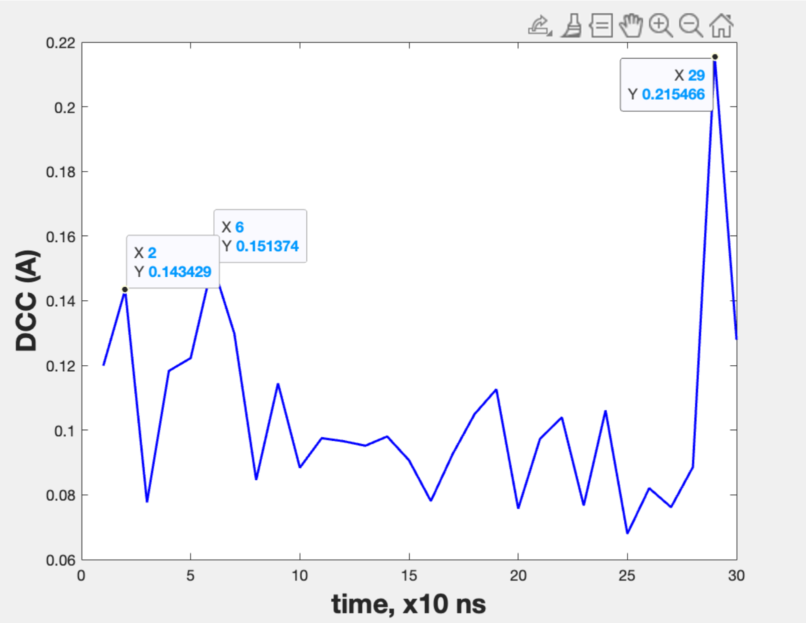
DCC (A)
time, x10 ns
X 29
Y 0.215466
X 6
Y 0.151374
X 2
Y 0.143429

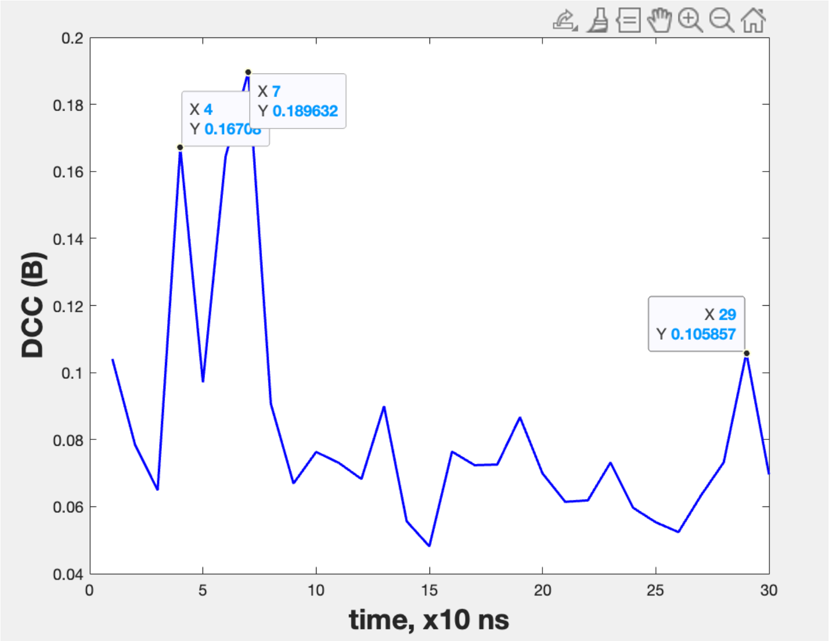
DCC (B)
time, x10 ns
X 7
Y 0.189632
X 4
X 29
Y 0.105857

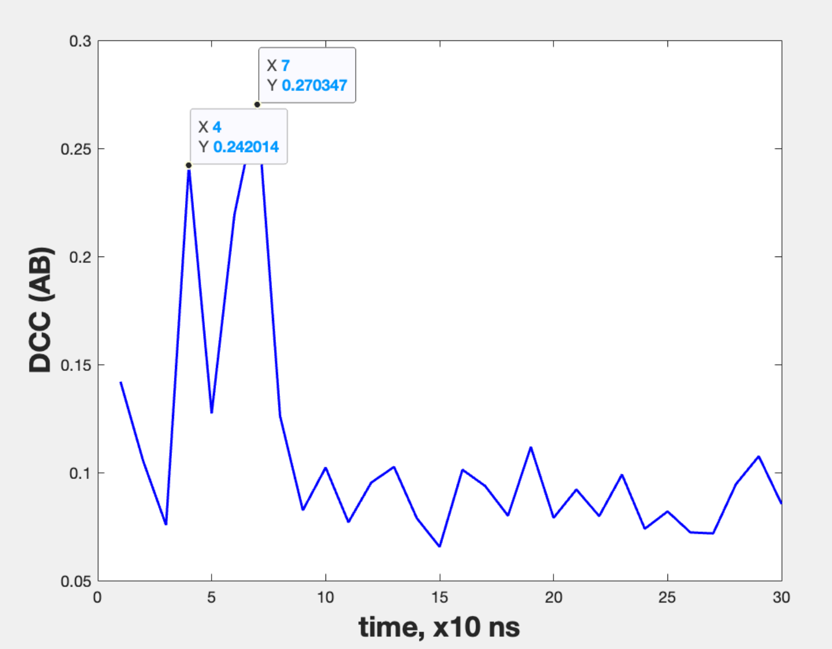
DCC (AB)
time, x10 ns
X 7
Y 0.270347
X 4
Y 0.242014

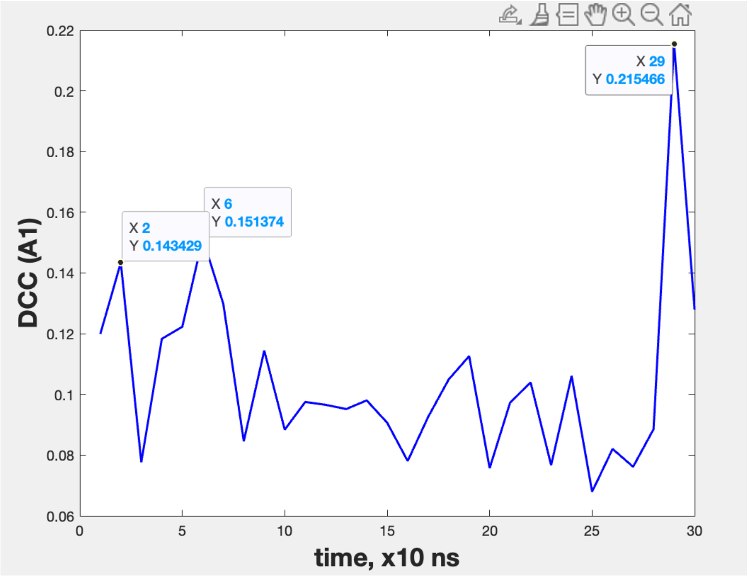
0.22
0.2
0.18
0.16
0.14
0.12
0.1
0.08
0.06
DCC (A1)
0
5
10
15
20
25
30
time, x10 ns
X 2
Y 0.143429
X 6
Y 0.151374
X 29
Y 0.215466

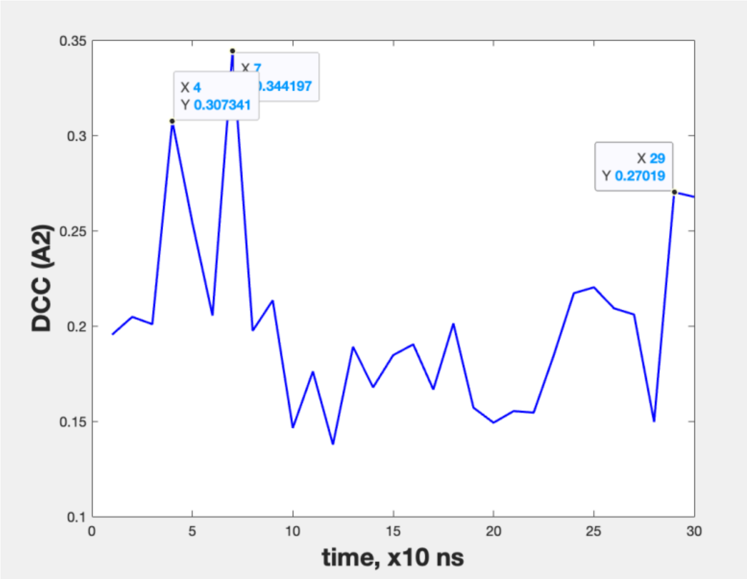
0.35
0.3
0.25
0.2
0.15
0.1
DCC (A2)
0
5
10
15
20
25
30
time, x10 ns
X 4
Y 0.307341
X 7
.344197
X 29
Y 0.27019

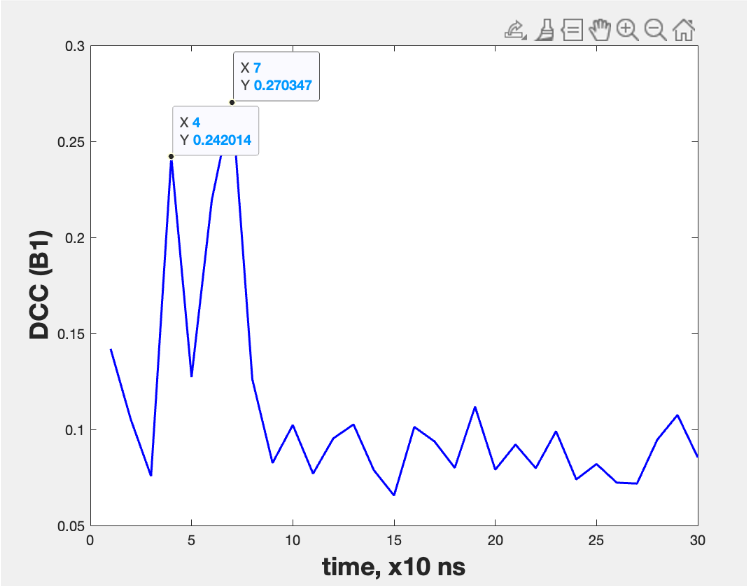
0.3
0.25
0.2
0.15
0.1
0.05
DCC (B1)
0
5
10
15
20
25
30
time, x10 ns
X 7
Y 0.270347
X 4
Y 0.242014

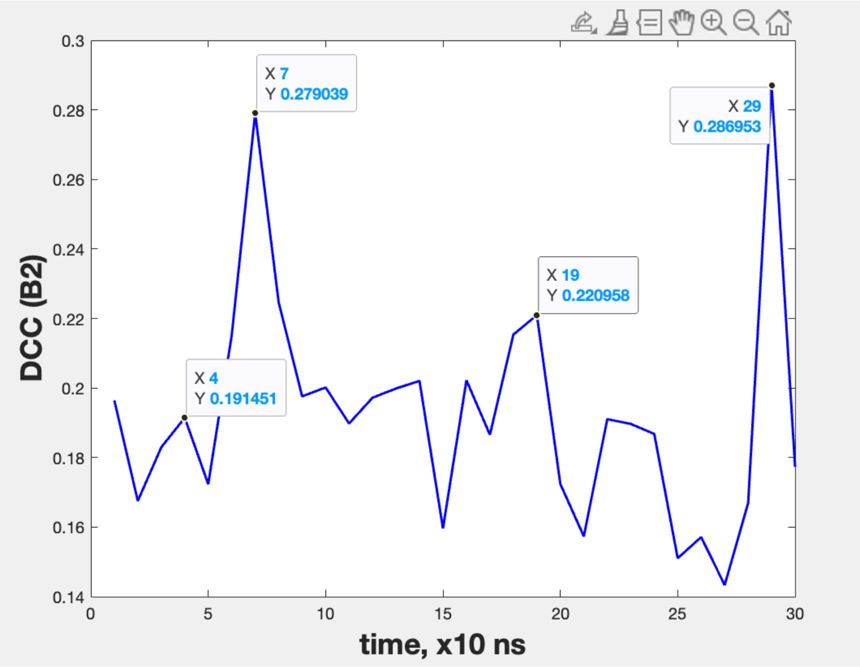
0.3
0.28
0.26
0.24
0.22
0.2
0.18
0.16
0.14
DCC (B2)
0
5
10
15
20
25
30
time, x10 ns
X 7
Y 0.279039
X 4
Y 0.191451
X 19
Y 0.220958
X 29
Y 0.286953

APO FORM

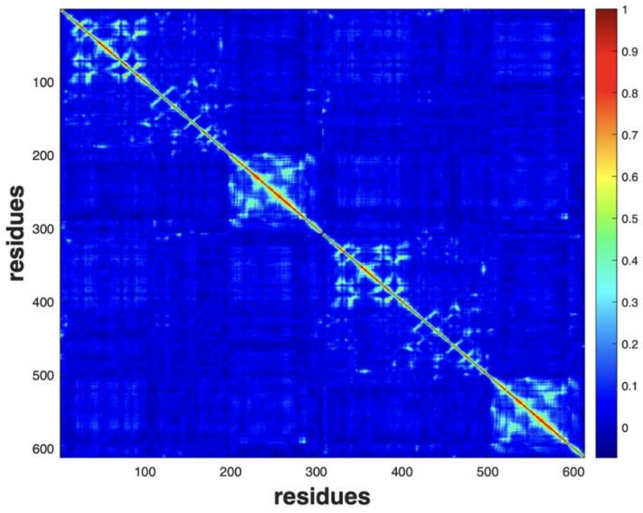

A

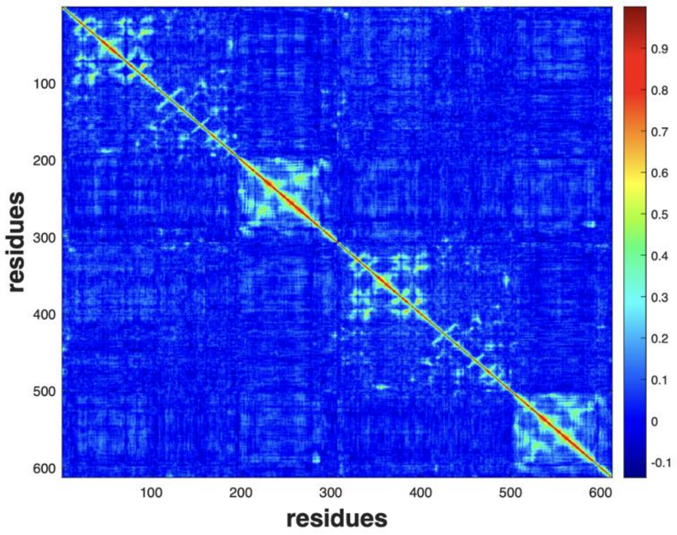

B

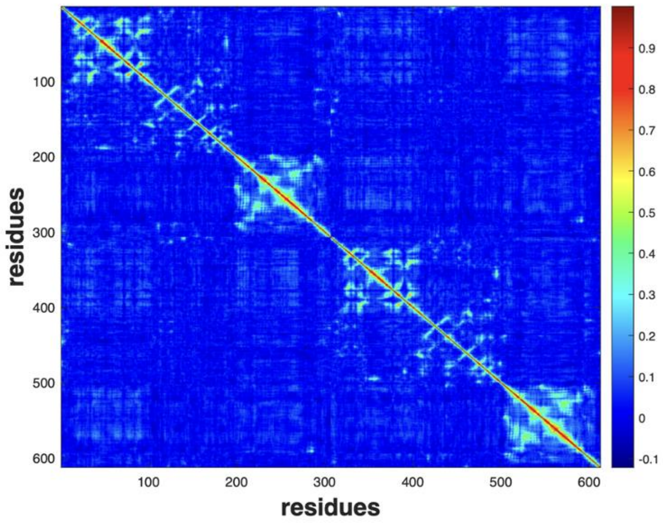

C

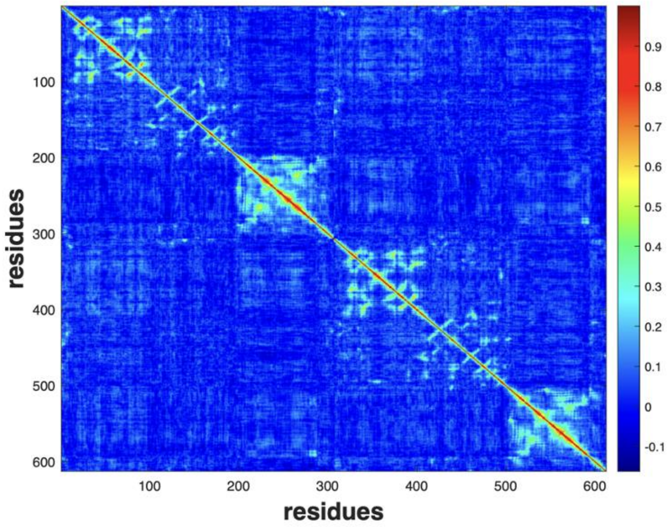

D
